# Durability Analysis of Formaldehyde/Solid Urban Waste Blends

**DOI:** 10.3390/polym11111838

**Published:** 2019-11-08

**Authors:** Francesca Ferrari, Raffaella Striani, Paolo Visconti, Carola Esposito Corcione, Antonio Greco

**Affiliations:** Department of Engineering for Innovation, University of Salento, 73100 Lecce, Italy; francesca.ferrari@unisalento.it (F.F.); raffaella.striani@unisalento.it (R.S.); paolo.visconti@unisalento.it (P.V.); antonio.greco@unisalento.it (A.G.)

**Keywords:** solid urban waste, formaldehyde, durability

## Abstract

Following the innovative research activity carried out in the framework of the POIROT (Italian acronym of dOmotic Platform for Inertization and tRaceability of Organic wasTe) Project, this work aims to optimize the composition of the blends between the organic fraction of municipal solid waste (OFMSW) and formaldehyde-based resins, in order to improve the durability properties. To this aim, in this work, commercial urea-formaldehyde and melamine-formaldehyde powder polymers have been proposed for the inertization of the OFMSW, according to the previous optimized OFMSW-transformation process. A preliminary study about the mechanical properties of the composite panels produced with the different resins was carried out by evaluating compressive, flexural, and tensile performances of the panels. Artificial weathering by cyclic (heating–cooling) and boiling tests were carried out and the mechanical properties were evaluated in order to assess the resistance of the panels to water and humidity. The melamine-formaldehyde based resin had the best performances also when subjected to the weathering tests and despite the higher content of resin in the composites, the panels produced with melamine-formaldehyde have the lowest values of release of formaldehyde minimizing their potential hazard level.

## 1. Introduction

In the last decade, municipal solid waste management has grown to be one of the main challenges of modern smart cities in the world. Any solution to improve the environmental sustainability represents a welcome candidate that can improve the quality of life for the population. In our previous research [1,2,3], we reported on the design and realization of an innovative prototype platform able to produce inertized and valorized panels, starting from the organic fraction of the municipal solid waste (OFMSW). The platform is managed by Arduino-based electronic sections for controlling process parameters and integrated with user recognition and a product traceability system based on radio frequency identification (RFID) technology. The POIROT (Italian acronym of dOmotic Platform for Inertization and tRaceability of Organic wasTe) prototype machinery implements a transformation process, constituted by different sequential steps for transforming the organic wastes into a fully inert material. In particular, the main purposes of the transformation process are:

To transform the organic material into bricks, which are inert from a bacteriological point of view and, thus, storable for a long time without any problems in domestic environments;

To modify the composition of the produced inert material by adding specific additives, for giving it appropriate physical and mechanical characteristics, aimed at targeting the specific application of the products, for efficient reuse and recycling;

To label by means of a RFID tag and to properly identify the products for allocating them into storage centers so that they can be recovered and then reutilized in a targeted manner, thanks to the possibility of identification and traceability given by the RFID technology.

Among the other thermos-setting resins, such as epoxy and polyurethane, urea-formaldehyde (UF) systems were previously selected for inertization of OFMSW [1,2], due to their distinct advantages, such as high crosslinking, water solubility, high strength, cost effectiveness, and rapid curing performance [4]. For its own specific characteristics formaldehyde is, in fact, employed for a wide range of applications. The large diffusion of formaldehyde-based materials is due to the high chemical activity and relative cheapness that allows the employment of formaldehyde in several industrial realities. The high bactericidal action is exploited in the medical sector for the conservation of biological material or for sterilizing and disinfecting in pharmacology. Formaldehyde is used in chemical synthesis in the production of detergents, soaps, shampoos, and other cosmetic products as well as for the manufacturing of building materials, such as plywood lacquers, coatings, or glues. Outside of industrial processes, it is also well known that formaldehyde exists naturally in some vegetables and fruits [5]. For instance, pears (38.7–60 mg/kg), grapes (22.4 mg/kg), potatoes (19.5 mg/kg), bananas (16.3 mg/kg), bulb vegetables (11.0 mg/kg), apples (6.3–22.3 mg/kg), carrots (6.7–10.0 mg/kg), and watermelons (9.2 mg/kg) are the aliments that contain the most amount of formaldehyde. Formaldehyde is also employed as a feed hygiene substance in feed for animals [6,7,8]. The food industry widely uses formaldehyde like food preservatives (identified as E240) especially in smoked food products [9]. The use of formaldehyde as a preservative for food is still an open issue in Europe [7,10], although concentrations equal to 2.5 g/kg are permitted in the United States [9,11]. On one side, people are exposed to small amounts of formaldehyde by eating food, on the other side, the greatest damage occurs by inhalation. Nowadays, automobile and aircraft exhaust emissions, natural gas, fossil fuels, waste incineration, and oil refineries are the major man-made sources of formaldehyde [12]. Formaldehyde is normally present in both indoor [13] and outdoor air [14]. Building materials can be significant emission sources of volatile organic compounds (VOCs), affecting high concentration levels in indoor environments [15,16]. The urea-formaldehyde foam for insulating (UFFI) the buildings, particularly diffused in the 1970s, was substituted, in recent years, by the urea-formaldehyde spray foam (UF) [14], that is dried to remove any volatile compounds, thus less formaldehyde would be expected to be released. A variety of products present in the home can be sources of formaldehydes release, among those more diffused are wood floor finishes, pressed-wood, and wood-based products containing UF resins, such as wallpaper, paints, cigarette smoke, cooking fumes, but also carpets or gypsum board. Due to their porosity, they absorb significant amounts of formaldehyde that could become trapped inside these materials and subsequently released over time in the indoor air. Despite the wide presence of formaldehyde in several materials, it has been classified as cytotoxic, a mutagen, and a human carcinogen by the International Agency for Research on Cancer [17,18,19,20]. For such reasons, it is very important to preventively note the risks for human health and the environment by evaluating the hazards in order to limit the exposure. The UF resins have been proven to be efficient, cost and time saving, materials for the inertization of OFMSW. In previous works [1,2,3], the pre-sterilized OFMSW is mixed with a given amount of UF and water for the production of a pourable slurry. The developed OFMSW-transformation process without any pressure application in the POIROT prototype machinery allows curing of the resin. This approach enables the resin to block the release of odors and percolates from the OFMSW, and at the same time produce low cost bricks or panels, which can be useful in different industrial and building applications.

On the other hand, the aforementioned issues associated with the use of UF-based systems suggest the possibility of replacing them, also in view of their poor water resistance, which can cause degradation of the properties and release of free formaldehyde. 

Therefore, this work is aimed at studying the suitableness of different formaldehyde systems (urea/formaldehyde and melamine/formaldehyde) for the inertization of the treated OFMSW. The choice of a proper matrix is mainly based on the durability of the produced panels, with particular emphasis on the resistance towards the effect of water, which can be detrimental for the mechanical properties, and at the same time can promote hydrolysis of the matrix, and consequent release of free formaldehyde.

## 2. Materials and Methods

Three resins, with decreasing formaldehyde content, were used in this work, in order to allow the intertization of the OFMSW, by producing composite panels with different performances:

1. Urea formaldehyde powder polymer, commercialized by Sadepan as SADECOL P 100N (Sadepan Chimica S.r.l, Viadana, Italy). It is supplied as fine powder/granules with defined grain size and a viscosity from 60 to 130 mPa*s at 20 °C. The resin is characterized by a formaldehyde content lower than 1% [21]. This resin is labelled as HUF (urea-formaldehyde with higher content of free formaldehyde);
2. Urea-formaldehyde powder polymer, commercialized by Sadepan as SADECOL P 410 (Sadepan Chimica S.r.l, Viadana, Italy). The resin is characterized by a formaldehyde content lower than 0.1% [22]. This resin is labeled as LUF (urea-formaldehyde with lower content of free formaldehyde);
3. Melamine-formaldehyde powder polymer, commercialized by Sadepan as SADECOL P 656 (Sadepan Chimica S.r.l, Viadana, Italy), obtained by condensation between melamine and formaldehyde, modified by addition of fillers, additives, and hardeners. The resin is characterized by a formaldehyde content lower than 0.1% [23]. This resin is labelled as MF (melamine–formaldehyde).

An amount of 10 wt% of a proper catalyst was added (Fast sad SD 10, supplied by Sadepan Chimica S.r.l, Viadana, Italy), in order to reduce the time and temperature of the curing process.

The OFMSW used was unsorted food waste collected according to standard Italian regulation. In particular, OFMSW was collected from a local dining room, and apart from food wastes, it contained small amounts (less than 0.5% in weight) of different soft wastes, such as tissues or napkins. 

The amount of resin was chosen as the lowest quantity able to reach a complete hardening after the cure (Table 1). Lower amounts involved a partial, or total, desegregation of the sample after the polymerization. Each sample is labelled according to the amount of the organic fraction of municipal solid waste (OFMSW), which is therefore, for three samples, equal to 80% (labelled as OF80), 70% (labelled as OF70) and 50% (labelled as OF50). However, it should be kept in mind that the OFMSW is actually made of about 70% by water, which, however, is removed by evaporation at the end of the curing process. Therefore, in Table 1, the amount of water and the amount of dry OFMSW are also reported. In addition, the composition of cured samples, in which water is completely removed, is reported. The three samples also differ by the type of matrix which was used. Sample OF80_HUF with 80% of wet OFMSW was obtained with the UF SADECOL P 100N matrix (above defined HUF resin) characterized by a higher free formaldehyde content, whereas sample OF70_LUF with 70% of wet OFMSW was obtained with the SADECOL P 410 matrix (above defined LUF resin) characterized by a lower content of free formaldehyde. Finally, the sample OF50_MF with 50% of wet OFMSW was obtained with the melamine-formaldehyde P 656 matrix (above defined MF resin).

Rheological analyses were carried out on a Rheometrics Ares rheometer (TA Instruments, New Castle, DE, USA) on the slurry produced with the different resins. Steady rate tests were carried out at 25 °C, varying the shear rate from 0.05 to 1 s^−1^, in order to evaluate the possible changes in viscosity due to the use of the different resins. In addition, dynamic temperature ramp tests were performed on all of the mixtures produced, in order to analyze the curing process of the samples during a heating scan from 25 to 120 °C with a heating rate of 5 °C/min, on a parallel geometry plate with a gap of 0.3 mm, constant oscillatory amplitude (1%), and frequency (1 Hz).

**Figure 1:**
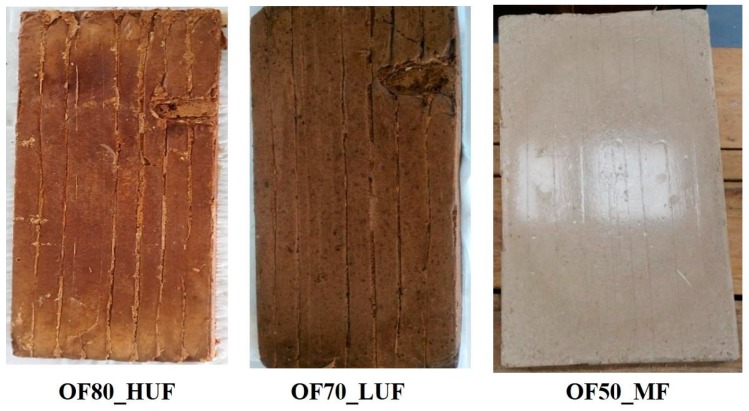
OFMSW-based panel production with different resins.

Several panels with the different resins of Table 1 (some of them shown in Figure 1) were produced by using the POIROT prototype machinery [1,3]. The latter prototype, besides the possibility to carry out a mechanical and thermal process for the transformation and valorization of the OFMSW, allows us to perform an electronic control aimed to check the correct operation of the different transformation processes. Additionally, a continuous diagnostic is ensured by means of appropriate measurement systems of the physical-chemical parameters related to each transformation phase of the conferred organic waste and of the intermediate and final waste water [3].

The dimensions of the produced panels are 400 mm × 200 mm × 30 mm, with an in-plane standard deviation of 3 mm and a through thickness standard deviation of 1 mm (Figure 1).

Compression, tensile, and flexural tests were performed according to, respectively, UNI EN 826 [24], UNI EN 319 [25], and UNI EN 310 [26] Italian standards that implement European directives on the cured panels for building applications. Before mechanical tests, all of the samples were weathered at RH 65%, 20 °C, up to constant weight. Referring to the standards, six samples were extracted from the panels with the following geometry:

UNI EN 826 (compression tests) [24]: sample dimensions equal to *t* × 50 mm × 50 mm, where *t* is the thickness of the panel (30 mm). Tests were carried out with a crosshead speed of 0.3 mm/min;

UNI EN 319 (tensile tests) [25]: sample dimensions equal to *L* × 50 mm × *t* , where *L* is 200 mm, corresponding to half of the length of the panel and *t* is the thickness of the panel (30 mm). The crosshead speed was chosen as 5 mm/min, in order to obtain broken samples in 60 s;

UNI EN 310 (flexural tests) [26]: sample dimensions equal to *t* × 50 mm × 20*t* , where *t* is the thickness of the panel (30 mm). The crosshead speed was chosen as 1.5 mm/min, in order to obtain broken samples in 60 s.

Afterwards, all the samples were subjected to cyclic boiling tests, in order to assess their resistance to water and humidity. In particular, according to UNI EN 321 [27], cyclic tests involved immersion in water for 70 h at 20 °C, followed by freezing at −18 °C for 24 h and heating at 70 °C for 70 h, and finally keeping them at room temperature for 4 h. The whole cycle is repeated three times. At the beginning and at the end of the test, the sample is weathered in a climatic chamber (at 20 °C and 65% RH) until constant weight is reached. 

Samples exposed to cyclic tests were then subjected to compression tests, according to UNI EN 826 standard.

Also, boiling tests were performed on samples extracted from the panels, by following the UNI EN 1087-1 standard [28]. Tests consisted of immersion of the samples in neutral water at 20 °C, followed by heating in an oven at 110 °C. After water boiling, the sample is held in the oven for 120 min. After cooling, differently from the cyclic tests, the sample is not weathered.

After boiling, all the samples were subjected to compression, tensile and flexural tests with the procedures described above.

For each test, six repetitions were performed. The six samples were extracted from three different panels, in order to account also for the change of the properties due to different batches of OFMSW.

Finally, the formaldehyde emission for each panel was assessed, according to the UNI EN 717-2 standard [29]. Tests, performed in external laboratories, consisted of placing samples of 400 mm × 50 mm × board thickness in a 4-liter cylindrical chamber with controlled temperature (60 ± 0.5 °C), relative humidity (RH ≤ 3%), air flow (60 ± 3 L/h) and pressure. Air is continuously passed through the chamber at 1 L/min over the test piece, whose edge was sealed with self-adhesive aluminum tape before testing. The determinations were made in duplicate using two different pieces and the actual formaldehyde value is the average of the two pieces after 4 h expressed in mg-HCHO/m^2^·h. The formaldehyde amount in the water is then determined photometrically by acetyl acetone spectrophotometric analysis. The determination is based on the Hantzsch reaction, in which aqueous formaldehyde reacts with ammonium ions and acetyl acetone to yield dia-cetyldihydrolutidine (DDL) [30].

## 3. Results and Discussion

**Figure 2:**
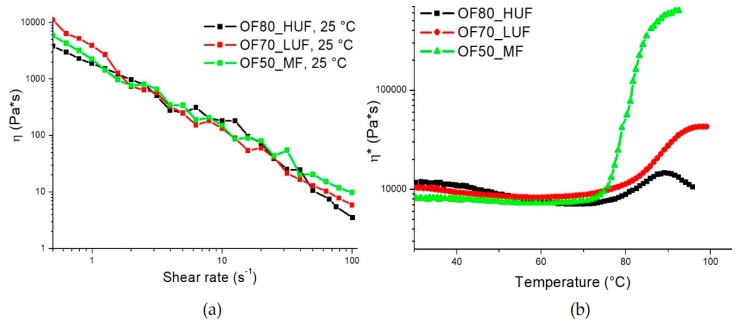
(**a**) Steady rate sweep tests at room temperature and (**b**) dynamic temperature ramp tests on OF80_HUF, OF70_LUF and OF50_MF samples.

In Figure 2a, the steady state viscosity is reported as a function of shear rate for the different formulations reported in Table 1. In order to attain a low viscosity of the slurry, necessary to allow its pressure-free processing in the POIROT prototype, the matrices used in this work required different amounts of water. In the developed process, the required amount of water was supplied by the OFMSW, which was composed of about 70% water and the remaining fraction (about 30%) is made, above all, of different types of lipids, carbohydrates, and proteins. Reducing the amount of water necessary to attain the same viscosity has the distinct advantage of reducing the porosity of the panels, which in turn involves better mechanical properties [3].

The reactivity of the different mixtures was assessed through dynamic rheological analyses. Results, reported in Figure 2b, show that all of the tested formulations are characterized by similar onset temperatures of curing (*T*_0_) (Table 2). On the other hand, as clearly shown in Figure 2b, the melamine/formaldehyde resin shows a much faster curing, as highlighted by the much higher slope of the curve (Table 2). Furthermore, the final viscosity (*η*_f_) that is reached with this sample is much higher than those of the other two blends. This result is related to the lower water content of the mixture, which allows lower reaction times and higher final viscosity values.

**Figure 3:**
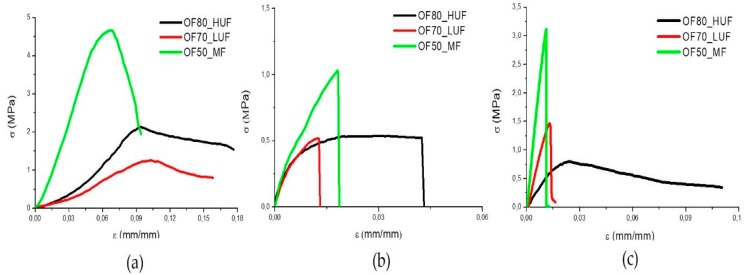
(**a**) Compression, (**b**) tensile, and (**c**) flexural tests on samples realized with different resins.

Typical stress-strain curves from compression, tensile, and flexural tests are reported in Figure 3a–c, respectively. The corresponding values for the strength (*σ*_R_), strain at break (*ε*_R_), and modulus (*E*) are reported in Table 3. The panels produced by the MF SADECOL P 656 resin are characterized by higher strength and elastic modulus in all of the loading conditions. This is due to the higher value of the density (*ρ*) of the panels, also reported in Table 3. In turn, the higher density is a direct consequence of the lower amount of water added in the slurry, which reduced the porosity of the panels. All of the samples show much better properties in compression than in tension. This is due to the brittle behavior of all of the panels and to the significant amount of porosity in each sample. As highlighted by the results of Table 3, all of the samples show, in flexural tests, an intermediate behavior between that measured in tension and in compression.

**Figure 4:**
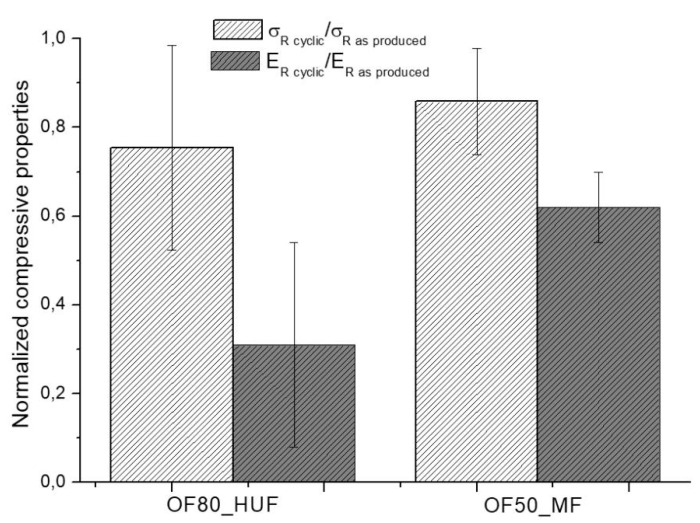
Normalized compressive tests after repeated heating-cooling cycles on OF80_HUF and OF50_MF samples.

Compression tests were then carried after subjecting samples to cyclic humidity weathering according to UNI EN 321 standard. Only the OF80_HUF and OF50_MF samples were tested, since OF70_LUF ones were completely broken after the heating–cooling cycles, as detailed in Section 2. Results, reported in Figure 4, indicate, for both systems, a decrease of about 20% in compressive strength. On the other hand, the compressive modulus was strongly affected by the heating-cooling cycles, with a reduction of about 40% for OF50_MF system and a sharp decrease of about 70% for OF80_HUF samples. Therefore, in accordance with the results in Table 3. Mechanical tests resultsthe analysis of the compressive behavior indicates better performances of OF50_MF samples even after cyclic tests.

Afterwards, all of the produced samples were subjected to boiling tests, according to UNI EN 1087-1. Once extracted, samples were tested in wet conditions with compressive, tensile, and flexural tests. 

**Figure 5:**
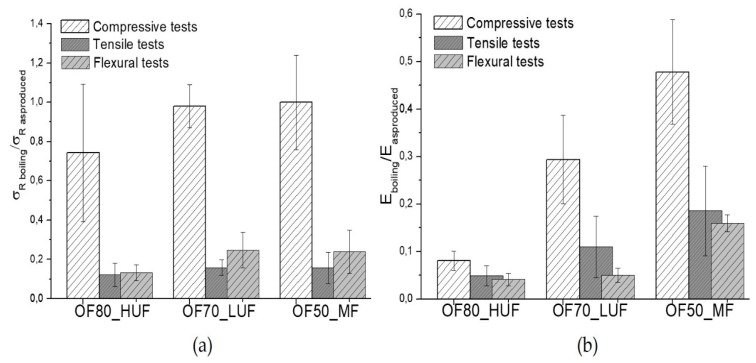
Mechanical response of OF80_HUF, OF70_LUF and OF50_MF samples after the boiling tests; ratio between strength of the samples after and before the boiling tests (**a**), ratio of samples’ modulus after and before the boiling tests (**b**).

All of the measured properties showed a significant decrease after the boiling tests. In particular, referring to compressive tests, the results of Figure 5a,b show a reduction of about 30% and 90% respectively for the strength and modulus of the OF80_HUF samples. Referring to compressive properties, the ratio between the strength of the OF50_MF sample after and before the aging are comparable to that of OF70_LUF sample, whereas the ratio of modulus after and before the boiling tests of OF50_MF sample is much higher than that of OF70_LUF sample.

Tensile and flexural properties are, in general, much more affected by the boiling tests compared to compression properties. In particular, a reduction of about 90% for the strength was found relative to the OF80_HUF sample, both in the flexural and tensile tests. The corresponding modulus decrease is about 95%. Therefore, even for boiling tests, the OF80_HUF sample showed the higher sensitivity.

The strength reduction for the OF70_LUF and OF50_MF samples are substantially equivalent both in tension and flexural tests. In both cases, a reduction of about 75–80% is found. However, the OF50_MF sample showed a better retention of the stiffness compared to the OF70_LUF sample, both in tension and flexural tests. Therefore, even in this case, better performances were reached with the OF50_MF composition.

**Figure 6:**
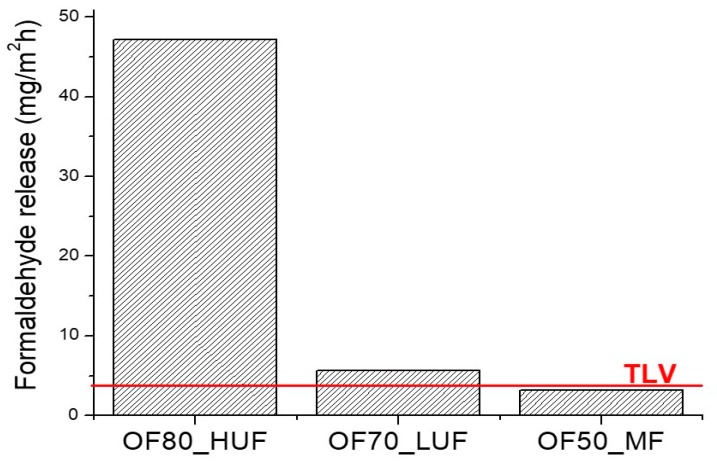
Formaldehyde release according to UNI EN 717-2 standard.

Formaldehyde release was evaluated according to the UNI EN 717-2 standard, in order to assess whether the formaldehyde emission of the produced panels is lower than the standard limits for manufactured housing at the time of sale, thus allowing a possible commercialization of the realized products. Results in Figure 6 indicate that only OF50_MF samples show a formaldehyde release lower than the threshold limit value (TLV), thus confirming that these samples comply with the UNI EN 717-2 standard. On the other hand, both OF80_HUF and OF70_LUF panels showed a higher formaldehyde release than the TLV, with a higher gap for OF80_HUF samples. This result is attributable to a higher water content of the slurry before the curing process. Higher moisture and water content implies higher formaldehyde emissions, either due to retention of dissolved formaldehyde, less effective cure, or higher hydrolysis rate. Therefore, a lower release could be attained by decreasing the water content of the mixtures before the curing process.

## 4. Conclusion

An innovative prototype machinery for the stabilization and valorization of the organic fraction of municipal solid waste (OFMSW) has been already assessed in the framework of the POIROT Project research activities, by using a urea–formaldehyde resin containing a formaldehyde content of less than 1 wt% [1,2]. In this paper, the possibility to substitute the previous resin with two different matrices containing a lower amount of free formaldehyde was evaluated. The composition of the blends between OFMSW and formaldehyde resins were, initially, optimized in order to improve the processability of the slurries. Attaining the same viscosity with the three different resins required different amounts of water, and therefore, different amounts of dry OFMWS. In particular, the melamine-formaldehyde SADECOL P 656 resin required the lower amount of water. The panels produced by the POIROT platform showed a density which is strictly correlated to the initial amount of water of the slurry. A lower porosity was found for samples requiring lower amount of water. 

As a consequence of this, the melamine–formaldehyde-based blend showed the best mechanical performances on the as-produced samples. However, even after artificial weathering cyclic heating–cooling or boiling tests, the melamine-formaldehyde based blend showed a better retention of the initial mechanical properties. In addition, the panels produced with melamine–formaldehyde showed the lowest values of formaldehyde release, evidencing the lowest hazard level. Starting from the results obtained in this work, the melamine–formaldehyde-based resin was selected as the more suitable matrix for the production of OFMSW-based composites with high mechanical and durability properties and low environmental impact, as achieved with the use of POIROT machinery.

## Figures and Tables

**Table 1:** Composition of the blends: OF80_HUF sample is a blend with 80% of wet OFMSW inertized in 20% of HUF resin, OF70_LUF refers to blend with 70% of wet OFMSW and 30% of LUF resin, finally OF50_MF is a blend with 50% of wet OFMSW and 50% of MF resin.

	Composition of the Slurry (wt%)			Composition of Cured Samples (wt%)	
Formulation	Water	Resin	Dry OFMSW	Resin	Dry OFMSW
**OF80_HUF**	54.9	21.6	23.5	48	52
**OF70_LUF**	47.9	31.6	20.5	60	40
**OF50_MF**	33.6	52	14.4	78	22

**Table 2:** Rheological data obtained from the curves of Figure 2 (*T*_0_ is the onset temperature of curing, *η*_f_ is the samples final viscosity).

Formulation	*T*_0_ (°C)	Slope (Pa*s/°C)	*η*_f_ (Pa*s)
**OF80_HUF**	74.2	0.02688	1.47E4
**OF70_LUF**	71.5	0.04939	4.41E4
**OF50_MF**	70.7	0.20397	6.83E6

**Table 3:** Mechanical tests results *(σ*_R_ is the samples strength, *ε*_R_ the strain at break, *E* the modulus and *ρ* the density).

Formulation	*σ*_R_ (MPa)	*ε*_R_ (mm/mm)	*E* (MPa)	*ρ* (g/cm^3^)
	**Compression Tests**			
**OF80_HUF**	1.75 ± 0.36	0.16 ± 8.5E-02	40.79 ± 3.48	0.76 ± 0.02
**OF70_LUF**	1.98 ± 0.72	0.27 ± 6E-03	26.61 ± 6.16	0.75 ± 0.04
**OF50_MF**	5.17 ± 1.04	0.12 ± 1.3E-02	86.48 ± 10.69	0.86 ± 0.05
	**Tension Tests**			
**OF80_HUF**	0.41 ± 0.13	0.07 ± 1.5E-02	79.14 ± 46.63	
**OF70_LUF**	0.44 ± 0.10	0.03 ± 1.4E-02	74.07 ± 20.84	
**OF50_MF**	1.02 ± 0.12	0.02 ± 3E-03	126.69 ± 15.87	
	**Flexural Tests**			
**OF80_HUF**	0.76 ± 0.24	0.07 ± 0.02	75.87 ± 21.51	
**OF70_LUF**	1.21 ± 0.17	0.01 ± 2E-03	118.27 ± 17.10	
**OF50_MF**	2.79 ± 0.41	0.01 ± 2E-03	236.43 ± 41.61	
